# Population Heterogeneity and Selection of Coronary Artery Disease Polygenic Scores

**DOI:** 10.3390/jpm14101025

**Published:** 2024-09-26

**Authors:** Carla Debernardi, Angelo Savoca, Alessandro De Gregorio, Elisabetta Casalone, Miriam Rosselli, Elton Jalis Herman, Cecilia Di Primio, Rosario Tumino, Sabina Sieri, Paolo Vineis, Salvatore Panico, Carlotta Sacerdote, Diego Ardissino, Rosanna Asselta, Giuseppe Matullo

**Affiliations:** 1 Genomic Variation, Complex Diseases and Population Medicine Unit, Department of Medical Sciences, University of Turin, 10126 Turin, Italy; carla.debernardi@unito.it (C.D.); angelo.savoca@unito.it (A.S.); alessandro.degregorio@unito.it (A.D.G.); elisabetta.casalone@unito.it (E.C.); miriam.rosselli@unito.it (M.R.); eltonjalisherman.eltonjalisherman@unito.it (E.J.H.); cecilia.diprimio@unito.it (C.D.P.); 2 Cancer Registry and Histopathology Unit, Azienda Ospedaliera “Civile-M.P. Arezzo”, 97100 Ragusa, Italy; rosario.tumino@asp.sr.it; 3 Epidemiology and Prevention Unit, Fondazione IRCCS Istituto Nazionale dei Tumori, 20100 Milan, Italy; sabina.sieri@istitutotumori.mi.it; 4 MRC-PHE Centre for Environment and Health, Imperial College London, London W12 0BZ, UK; p.vineis@imperial.ac.uk; 5 Department of Clinical and Experimental Medicine, University Federico II, 80100 Naples, Italy; salvatore.panico@unina.it; 6 Piedmont Reference Centre for Epidemiology and Cancer Prevention (CPO Piemonte), 10126 Turin, Italy; carlotta.sacerdote@cpo.it; 7 Cardiology Department, Azienda Ospedaliero-Universitaria of Parma, 43100 Parma, Italy; diego.ardissino@unipr.it; 8 Department of Medicine and Surgery, University of Parma, 43100 Parma, Italy; 9 Department of Biomedical Sciences, Humanitas University, Pieve Emanuele, 20072 Milan, Italy; rosanna.asselta@hunimed.eu; 10 IRCCS Humanitas Research Hospital, Rozzano, 20089 Milan, Italy; 11 Medical Genetic Service, Città della Salute e della Scienza, 10126 Turin, Italy

**Keywords:** polygenic risk score (PRS), coronary artery disease (CAD), disease genetic risk, population heterogeneity

## Abstract

Background/Objectives: The identification of coronary artery disease (CAD) high-risk individuals is a major clinical need for timely diagnosis and intervention. Many different polygenic scores (PGSs) for CAD risk are available today to estimate the genetic risk. It is necessary to carefully choose the score to use, in particular for studies on populations, which are not adequately represented in the large datasets of European biobanks, such as the Italian one. This work aimed to analyze which PGS had the best performance within the Italian population. Methods: We used two Italian independent cohorts: the EPICOR case–control study (576 individuals) and the Atherosclerosis, Thrombosis, and Vascular Biology (ATVB) Italian study (3359 individuals). We evaluated 266 PGS for cardiovascular disease risk from the PGS Catalog, selecting 51 for CAD. Results: Distributions between patients and controls were significantly different for 49 scores (*p*-value < 0.01). Only five PGS have been trained and tested for the European population specifically. PGS003727 demonstrated to be the most accurate when evaluated independently (EPICOR AUC = 0.68; ATVB AUC = 0.80). Taking into account the conventional CAD risk factors further enhanced the performance of the model, particularly in the ATVB study (*p*-value = 0.0003). Conclusions: European CAD PGS could have different risk estimates in peculiar populations, such as the Italian one, as well as in various geographical macro areas. Therefore, further evaluation is recommended for clinical applicability.

## 1. Introduction

Coronary artery disease (CAD) is a metabolic-related disorder responsible for the most serious causes of morbidity and mortality in Western countries and around the world [1]. CAD has high heritability (50–60%) [2], however, as of today, genetic testing is not readily used in clinical practice, except in the context of Mendelian diseases such as familial hypercholesterolemia or to stratify patients for therapeutic maneuvers (e.g., PCSK9) [3].

Genome-wide association studies (GWASs) have shown that hundreds of genetic variants influence complex human phenotypes. These studies have identified many loci that are associated with CAD susceptibility, mostly in populations of European ancestry [4]. Typically, each of these variants has a small effect size, i.e., individually, they contribute to a minor fraction of the phenotypic variation, but when considered together, they account for a relatively large fraction of the heritability [5]. This observation opened up the possibility to use genetic variants distributed across the genome to calculate genetic risk through polygenic scores (PGSs) and predict an individual’s risk of developing diseases.

Many PGS were developed and demonstrated to be predictive of common complex traits and diseases [6,7,8], defining an elevated risk over their lifetime or at an earlier age for people with high scores. Existing risk-prediction models based on traditional risk factors could be improved by incorporating individual risk scores [9]. In some cases, PGSs may be the most informative risk factors in presymptomatic individuals, as genetic scores could be independent of the family history for some disease [10]. Despite this, the interpretation of these scores is challenging due to the wide variability, both in terms of the number of variants included and their effect on disease risk.

The PGS Catalog (https://www.pgscatalog.org (accessed on 11 October 2023)) is an open resource where researchers can upload and share published PGSs and metadata that could allow for ease of access as well as promoting reproducibility [11]. Numerous studies have shown that most PGSs are based on the European population and reach a lower performance when applied to populations with non-European ancestry [12,13]. This loss of accuracy is primarily driven by differences in linkage disequilibrium (LD) [14], allele frequency (including population-specific SNPs) [15,16], and causal effect size [13], although differences in heritability also play a minor role [15].

Choosing which PGS to use for a specific study is not trivial; the above considerations must be taken into account if the study population has particular genetic characteristics.

The Italian population has greater internal genomic variability compared to other European countries, which is the result of Italy’s geographical topography and history [17,18]. A correlation between genetic and geographic distance in Europe and also in Italy was found, where a clear cline between Southern Italians and other Europeans can be observed [19,20,21]. To enable clinical applications of these scores, it is mandatory to perform a more detailed analysis of specific populations, in particular for countries with a significant genetic heterogeneity, such as Italy.

In this study, we aimed to investigate the predictive power and transferability of different PGS (extracted from the PGS Catalog), in particular those based on European ancestry in the Italian population, to examine whether the individual PGSs improve risk prediction beyond conventional risk factors and support practical use in the clinic.

## 2. Materials and Methods

### 2.1. Population Characteristics

We evaluated the CAD predictive value of different polygenic scores (PGSs) in two different Italian studies. The first study is the EPICOR Study [22], a case-cohort study nested within the EPIC–Italy prospective cohort (286 cases and 290 controls) recruited among those enrolled in the EPICOR study [23]. All EPICOR cases developed myocardial infarction (MI) after recruitment (average time to diagnosis 6.90 years).

EPICOR participants were enrolled in four different Italian centers: Varese (n = 218), Turin (n = 300), Ragusa (n = 44), and Naples (n = 22). A large series of healthy adults were enrolled to identify all newly diagnosed cases of cancer and other relevant chronic diseases occurring after the date of enrollment and to study the risks associated with dietary and lifestyle habits reported at baseline. Cardiovascular events were identified at cohort follow-up from hospital discharge databases. The participants were contacted by media advertising and through nonprofit organizations such as blood donors, consumer groups, and patient aid associations.

The second study was composed of 3359 subjects; 1691 had the first MI at an age of <56 years, and 1668 were disease-free individuals; all were enrolled in by the Atherosclerosis, Thrombosis, and Vascular Biology Italian Study Group (ATVB) in the frame of a research project on early-onset myocardial infarction [24,25]. The main CAD endpoints were defined as either fatal or non-fatal first MI in both cohorts. CAD risk factors considered were conventional CAD risk factors (age, sex, body mass index—BMI, diabetes, and diastolic and systolic blood pressure) and behavioral (smoking status, alcohol consumption, and coffee consumption). We also included anthropometric measures (height and weight) and biochemical parameters (plasma glucose, LDL cholesterol, HDL cholesterol, and triglycerides).

To take into account the population structure in the analysis, we used the first four genetic principal components (PCA; see more details in Statistical Analyses and Supplementary Materials).

In the ATVB study, individuals were classified into four different geographic macro-areas: 2030 in the North, 420 in the Center, 744 in the South, and 68 in Sardinia [17,18,19,20,21,22,23,24,25,26].

The study protocols were approved by the Ethics Committee of recruiting centers. All participants signed an informed consent form for the use of their clinical and genetic data, in accordance with relevant guidelines and regulations.

### 2.2. PGS Selection

The PGS Catalog (v20240318) contains 266 polygenic scores for the “Cardiovascular Disease” category (Supplementary Materials), of which 51 are specific for CAD (EFO_0001645).

The polygenic risk scores for all individuals in the two studies were calculated through the Michigan Imputation Server as for the ancestry estimation and principal component analyses to verify their geographical origin (Supplementary Materials).

Only five of them were developed and evaluated on European sample sets: PGS000010 [27], PGS000329 [28], PGS001355 [29], PGS003727 [30], and PGS004595 [31].

### 2.3. Statistical Analysis

Downstream analyses were performed using R Statistical Software (v4.1.2; R Core Team 2021). We used the Kolmogorov–Smirnov test to compare the score distribution, and the Mann–Whitney and Fisher test were applied to determine differences between groups. Pearson correlation was used to measure linear correlation between variables. Odds ratios (ORs) with 95% CI were calculated using as a reference all individuals with a score lower than the percentile taken into consideration, and they were adjusted for age, sex, and BMI. Linear generalized models were used to build models to evaluate the implementation of PRS in stratifying patients according to their CAD risk, splitting the dataset in training (80%) with an internal 10-fold validation repeated 10 times and test set (20%), and selecting features through the BORUTA Machine Learning algorithm. The DeLong Test for the comparison of the areas under the ROC curves (AUC). *p*-value < 0.05 was considered statistically significant.

## 3. Results

### 3.1. Population Characteristics and Quality Control

The datasets selected from the EPICOR and ATVB studies for our study comprised 576 and 3359 genotyped samples, respectively. There are 7,080,677 imputed variants for EPICOR and 7,263,254 for ATVB, each with an imputation quality score (Rsq) above 0.6. Through our quality control process, several exclusions were made: 5 EPICOR and 97 ATVB samples were classified as non-Italian based on principal component analysis (PCA). In terms of variant quality, we excluded 796 variants from the EPICOR dataset and 1338 from ATVB that had a low call rate or allele mismatch. Within these cohorts, 286 (EPICOR) and 1691 (ATVB) individuals developed an MI, and Table 1 summarizes all the major demographic and clinical characteristics of the two studies.

As expected, the age between the two groups was significantly different (*p*-value < 0.0001), with the ATVB participants being younger (mean age 40, SD ± 4.9) than the EPICOR ones (mean age 53, SD ± 7.4). Indeed, the ATVB participants were enrolled to study juvenile myocardial infarction (MI), with patients suffering a first event before 57 years, while in the EPICOR study only 191 individuals developed a MI before 57 years.

In both studies, men were prevalent (EPICOR: 206 females and 387 males; ATVB: 2972 males and only 387 females), whereas the number of individuals that manifested a CAD event and disease-free participants were balanced in both studies for sex and age (Table 1).

In the EPICOR study, treated diabetic patients were excluded from the analyses to prevent an important association with decreases in dietary fat and cholesterol, whereas in the ATVB cohort it was not an exclusion criterion.

In the ATVB study, individuals from different regions of Italy were enrolled; in particular, both studies had the majority of individuals from Northern Italy (EPICOR: 518, 90%; ATVB: 2030, 61%).

There are some differences for CAD risk factors in the different areas. In the group from the north of the Italian peninsula, the percentage of cases is lower also due to the total number (Supplementary Materials: Table S4). Individuals from Northern Italy appear to have slightly lower triglyceride and blood sugar levels than other groups, and there are fewer smokers. In other geographical areas, the percentage of cases is around 60%. The only region with slightly different values is Sardinia, where the averages of BMI, total cholesterol, and LDL are lower than in the other areas. These differences could be due to the higher percentage of women present in this group. Regarding age and blood pressure, values do not appear to differ between the groups.

In addition, Michigan Imputation Server (Supplementary Materials) estimated all individuals to have European (EUR) genetic ancestry through PCA.

### 3.2. CAD Risk Predictive Power of PGS

We evaluated 266 cardiovascular disease polygenic scores, and 104 (39%) showed good performance in both studies (*p*-value < 0.05) using different types of tests. The Kolmogorov–Smirnov test was used to assess if cases and controls PRS had significant distributions, while the Mann–Whitney test was intended to compare differences between two independent groups and the linear generalized model to verify the association between developing a CAD event and the polygenic score is statistically significant. A comparison of the performance of evaluated models is depicted in Supplementary Materials; then, we selected 49 PGS specific for coronary artery disease (EFO_code 0001645), and from these, 5 were specifically built for European people, as summarized in Table 2: PGS000010, PGS000329, PSG001355, PGS003727, and PGS004595 (Supplementary Materials: Figure S1).

They are composed of a different number of SNPs, from the little PGS000010 (27 variants) to the larger PGS00329 (6423165 variants). The authors selected the variants with different methods; the smaller PGS000010 and PGS4595 (164 variants) were built using the significant variants from genome-wide studies. PGS000329 and PGS003727 (1125113 variants) were built with a larger selection by LDpred and LDpred2 methods, while PGS001355 (2994055 variants) comes from an original method of the authors called ANNOPRED.

The size of the population used is fundamental for estimating the weights of a polygenic score; PGS000010 came from a sample of 86,995 individuals, while PGS004595 came from 773,268 samples (Supplementary Materials: Table S1).

PSG001355 and PGS003727 were covered for more than 99% of the SNPs in both our studies; also, PGS000329 (97.4%) had a high coverage, while PGS000010 (81.5%) and PGS004595 (77.4%) had a lower coverage (Supplementary Materials: Tables S2 and S3).

The scores derived from the five PGSs did not show a significant correlation with patients’ characteristics. In particular, none of the scores had a correlation index > 0.75 with any of the characteristics that are highly correlated to CAD; these results underline the independent contribution of genetics to the evaluation of CAD risk.

**Figure 1:**
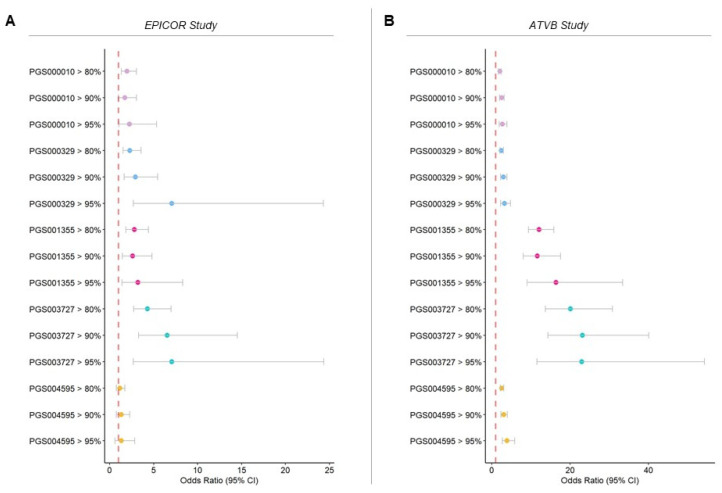
CAD risk for individuals with a high polygenic score. Odds ratio adjusted for age, sex, and BMI for the individuals whose score falls above the 80th, 90th and 95th percentiles, including the related 95% CI in the EPICOR (**A**) and ATVB studies (**B**). The comparison is with all the other individuals for each study.

All the scores showed an increased risk for individuals whose scores fall above the 80th percentile, which is enhanced for people with higher scores (Figure 1, Supplementary Materials: Tables S2 and S3). This analysis showed the risk to develop a CAD event for those with a high score in comparison with those with a lower score, dividing the PGS distribution into percentiles.

PGS000329 and PGS003727 achieved the highest risk for individuals with the scores in the last fifth percentile (odds ratio (OR) = 7.1, 95% CI = 2.7–24.4) in the EPICOR study (Figure 1, Supplementary Materials: Table S2). PGS001355 and PGS003727 achieved the highest risk for individuals with the scores in the last 5% percentile (PGS001355 OR = 16.4 (95% CI = 9.0–33.4), PGS003727 OR = 22.93 (95% CI = 11.52–54.32)) in the ATVB study (Figure 1, Supplementary Materials: Table S3).

**Figure 2:**
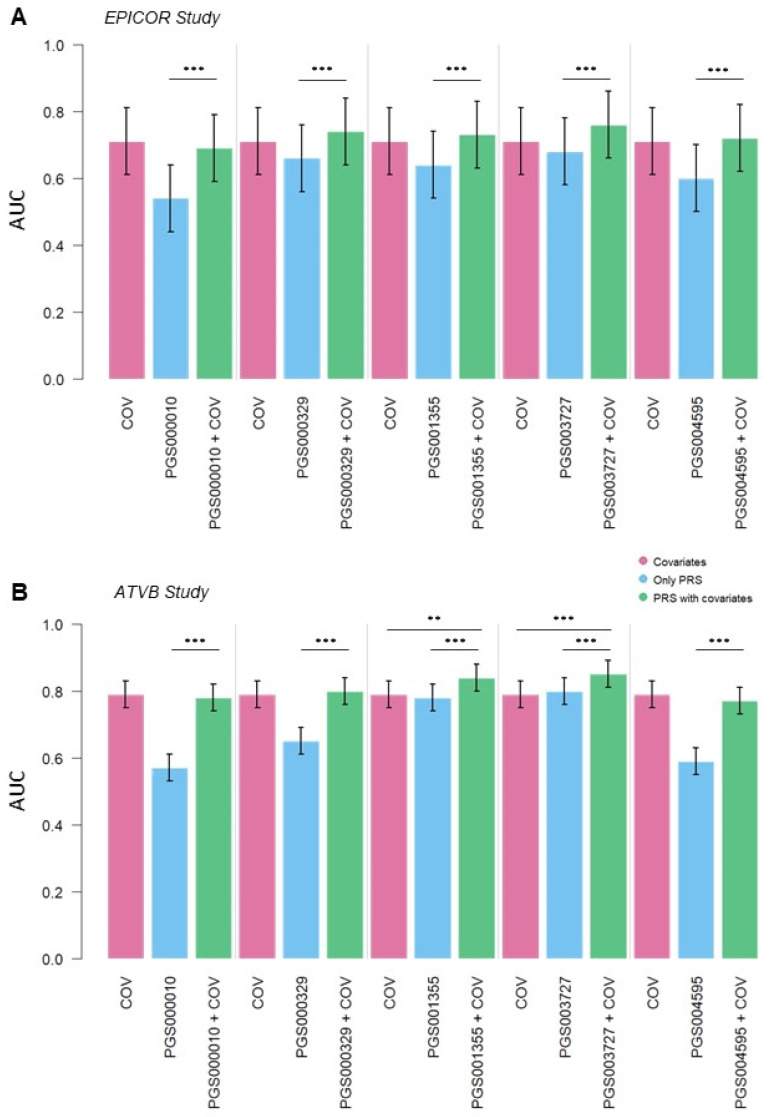
PGS model comparison. Graph A showed the AUC and 95% CI of ROC curves built with different models in the EPICOR study (**A**) and the ATVB study (**B**). In dark pink, models built only with covariates selected through the BORUTA algorithm; in light blue, models built using only PGS; and in green, the models that combine each PGS with the covariates. *** indicate a DeLong’s test *p*-value < 0.0001; ** indicate a DeLong’s test *p*-value < 0.001.

The performance of these scores was tested using them alone in a risk prediction model and adding the PGSs to a model containing clinical CAD risk factors. PGS000010 and PGS004595 showed the lowest value of area under the ROC (receiver operating characteristic) curve (AUC) (Figure 2, Supplementary Materials: Tables S2 and S3). PGS000329 had a better performance in EPICOR than in ATVB, as PGS001355 had a much better performance in ATVB than in EPICOR (Figure 2 and Supplementary Materials: Tables S2 and S3).

PGS003727 reached the highest area under the ROC (receiver operating characteristic) curve (AUC) value of 0.68 (95% confidence interval (CI) = 0.59–0.78) in EPICOR and 0.80 (95% CI = 0.77–0.84) in ATVB.

The classification performance of PGS003727 demonstrated a notable improvement, reaching an AUC of 0.76 (95% CI = 0.66–0.85) in EPICOR and of 0.85 (95% CI = 0.81–0.88) in ATVB when accounting for conventional CAD risk factors selected through the BORUTA algorithm: age, sex, diabetes, smoking status, high-density lipoprotein (HDL), low-density lipoprotein (LDL), and hypertension (Figure 2, Supplementary Materials: Tables S2 and S3). Additionally, there was a significant difference in performance between the model incorporating only risk factors selected through the BORUTA algorithm and the model with PGS003727 adjusted for risk factors selected through the BORUTA (DeLong’s test *p*-value = 0.0003) in the ATVB study. Moreover, PGS001355 added a significant improvement in covariates model performance (DeLong’s test *p*-value = 0.002) (Figure 2, Supplementary Materials: Tables S2 and S3).

### 3.3. Differences in the PRS among Different Geographical Italian Macro-Areas

We analyzed the distribution of the scores of the five different PGS within the ATVB study, with the goal to investigate if the scores change according to the different geographical origins within Italy (Supplementary Materials: Figure S2).

**Figure 3:**
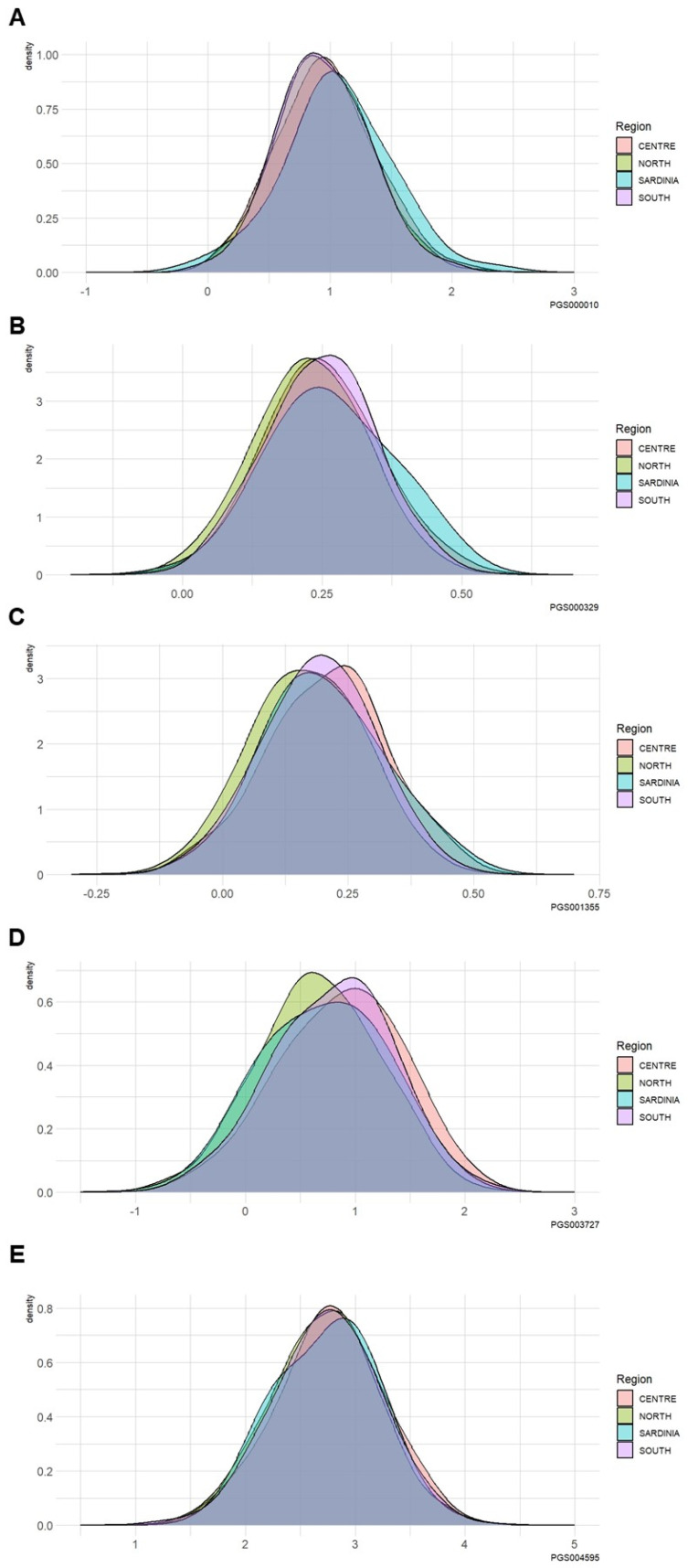
PGS distribution in Italian geographical macro-areas. Each plot shows PGS distributions: PGS000010 (**A**), PGS000329 (**B**), PGS001355 (**C**), PGS003727 (**D**), and PGS004595 (**E**) in the Italian macro-areas displayed with different colors.

The PGS004595 distributions were well overlapped for the different macro-areas, but the other scores showed some differences (Figure 3). Sardinian individuals showed higher scores than those from other Italian macro-areas for PGS000010, PGS00329, and PGS001355, while individuals from the north of Italy showed lower scores as concern scores for PGS000329, PGS001355, and PGS003727. In particular, PGS003727 score distributions differed considerably depending on the macro-area (Figure 3, Table 2).

Individuals that developed a CAD event had a score distribution significantly different from the controls in all macro-areas when considering PGS000329, PGS001355, and PGS003727 (Supplementary Materials: Figure S3). PGS000010 and PGS004595 did not show different score distributions for cases and controls for Sardinian individuals. Similarly, PGS000010 did not show different score distributions for cases and controls from Central Italy (Supplementary Materials: Figure S3).

We also investigated if, for each score, there were different distributions between males and females (Table 2). The Kolmogorov–Smirnov test showed no differences caused by sex for any of the PGS. In addition, PGS stratified by sex in different macro-areas showed no difference in the distribution (Table 2).

Despite differences in score distributions, the classification performance of PGS000329 had an AUC = 0.66 in all macro-areas except in Sardinia, where the AUC = 0.62 (95% CI = 0.27–0.98). PGS000010 showed the lowest performance, particularly in the individuals from the South of Italy (AUC = 0.55, 95% CI = 0.46–0.64). PGS003727 reached the highest value of AUC in all the macro-areas (Supplementary Materials: Table S5), followed by PGS001355.

## 4. Discussion

Polygenic risk scores are simple and relatively inexpensive; their implementation in the clinical setting holds a great promise. PRS could be a preventive tool, which could allow individuals with a genetic predisposition to have healthier behaviors and to do prevention from a young age.

For CAD, in particular, early identification of at-high-risk individuals could lead to simple yet extremely efficacious therapeutic interventions (e.g., statins and aspirin) or to simple lifestyle change interventions (e.g., physical exercise, diet modifications, etc.), allowing a reduction in the cost of healthcare, especially of rehabilitation care, and saving resources.

Importantly, PRS represent the combination of many risk factors rather than one single pathway that leads to disease. Individuals with a high genetic CAD risk could benefit from broad treatment and risk reduction strategies.

We tested all developed CAD PGS in two Italian studies recruited previously to understand which of them had the best performance on the Italian population.

Then, we selected scores from the PGS Catalog that were trained and tested on a 100% European population, checking that the individuals in both studies were traceable to European ancestry via PCA.

In our samples, the distributions for all PGS models followed a similar pattern, with the ATVB study showing lower *p*-values than EPICOR ones. This difference could be due to the different number of individuals in the two studies. Moreover, ATVB individuals are younger than in EPICOR. Therefore, the genetic component could have more impact on the CAD risk than in the EPICOR studies, in which individuals are 13 years older and risk factors linked to poor lifestyle habits may have had more influence on the CAD risk.

Evaluating the discrimination and prediction power of the PGS, both pre-diagnostic CAD patients from the EPICOR study and myocardial infarction patients from the ATVB study showed higher scores than their matched controls. The OR between the groups of patients whose score fell in the highest 20th percentile was significantly increased in both studies. In the ATVB study, in which the number of individuals was higher, we could notice that the risk increased significantly in the highest decile and even more in the last 5th percentile. With the exception of PGS003727, for which considerable ORs and extensive confidence intervals are reached, individuals with a score in the highest 5th percentile have a slightly lower OR than those in the last decile (Supplementary Materials: Table S3). Also, PGS001355 showed relatively high ORs for ATVB patients, but it maintains a growing trend as the score increases (Figure 2 and Supplementary Materials: Tables S2 and S3). This seems to indicate a significant increase in discrimination accuracy of the three different scores. We also observed that in the EPICOR study, individuals with more episodes had higher scores, suggesting that a high score could be related to a risk of a more severe disease.

Another cause of the above-mentioned differences could be the genetic composition of the Italian population. As previous studies reported, there is a certain degree of genetic heterogeneity between Northern and Southern Italy [17]. In particular, participants from the Sicily [32,33] and Sardinia [18,19,20,21,22,23,24,25,26,27,28,29,30,31,32,33,34] islands were genetically more different from the rest of the peninsula and with Middle Eastern and African influences. It is well known that predictive performance of European ancestry-derived PGS is lower in non-European ancestry samples [12,35], showing the influence of ancestry and genetic distance on genomic prediction [36,37]. Several studies on particular populations suggest including variants discovered from diverse populations to improve polygenic risk score transferability [38,39]. However, studies on populations with higher internal heterogeneity are lacking.

Along this line, we carried out stratified analyses for four geographical macro-areas (North, Center, South, and Sardinia) of the Italian peninsula. The results clearly show that the performances of the PGS are not equivalent in the different macro areas. The PGS000010 cannot discriminate between cases and controls from Central Italy and Sardinia. The well-known genetic differences that characterize the Sardinian population also make PGS004595 less efficient. The lowest performance of these two PGSs in Sardinian people could be due to the low number of the included variants.

Genetic heterogeneity is also well demonstrated by the different distributions of PGS000329 scores and the PGS001355 scores, which are significantly different between North, Center, and South.

To determine which of the three PGSs had the best performance in our population, we built a prediction model based on our studies, using a feature selection algorithm to identify the set of covariates with the highest predictive value. The selected features represent the most common CAD risk factors: lipid levels, smoking status, high blood pressure, and diabetes [40]. We noticed an increase in the predictive power for all five PGSs in both studies. Only the model with PGS001355 and PGS003727 showed a significant improvement in the prediction for the ATVB study.

Our study raises a few interesting issues. The first important consideration regards the number of variants in PGSs. We showed that the PGS000010 (27 variants) and PGS004595 (164 variants) had slightly lower performances compared to the other scores calculated with millions of SNPs. Due to such a small number of variants used, they are also the two scores with the lowest coverage (PGS0000010 = 81.5%, PGS0004595 = 77.4%). It is possible that by managing to use 100% of the variants selected by these scores, their performance can be improved. Moreover, PGS000329 includes almost twice the number of variants of PGS001355 and twice the number of variants of PGS003727, including more than 81% of all the other PGS variants, without showing the same high performances.

Interestingly, PGS003727, which showed a great performance in both studies, only shares 57% of variants with PGS001355 and contains a low percentage of variants with the smaller PGS000010 (55%) and PGS4595 (53%). These results seem to suggest that choosing the variants to include in a PGS more carefully can improve the results without being too restrictive.

Nonetheless, the predicting value of each variant was treated differently for half of these, showing an effect weight with the opposite sign. This could happen because of the different methods used to develop the scores [41]. It is also true that the majority of these variants have a small effect and typically correspond to a small fraction of truly associated variants, meaning that they have limited predictive power [42]. In any case, it must be taken into account that, by considerably increasing the number of SNPs, biological interpretation becomes difficult, as well as the understanding of the major pathways involved and the potential biological therapeutic targets. Furthermore, it is important to consider potential differences due to internal population genetic heterogeneity and interindividual local ancestry influencing the final PGS performance.

Choosing the right PGS for a study population is not trivial for all the aforementioned reasons. Furthermore, we have seen that the same individual can have different scores and very different risk estimates depending on the PGS used.

The ethical implications of using PGS in clinical practice must not be forgotten, particularly concerning health disparities, genetic discrimination, and, above all, patients’ perceptions of this type of information and the possible psychological consequences (e.g., anxiety). For this reason, studies are underway to evaluate the efficacy and feasibility of CAD PRS [43].

To the best of our knowledge, there are no publications describing a specific CAD polygenic risk score for the Italian population, nor results validating and comparing European PGS within the Italian population.

## 5. Conclusions

Our data suggest that choosing a CAD PGS based on the European population can perform well on the Italian population, but with some evaluations, because performances can vary greatly and may not be the same for each cohort, as for PGS000329, which seems to perform better in EPICOR than in the ATVB study.

In general, we should expect that a PGS based on a few hundreds of variants with a higher impact on CAD, specific for the Italian population, and taking into account more accurately local ancestry could have better prediction power.

Further studies taking into account internal population genetic heterogeneity and inter-individual local ancestry are needed, but our results move a step forward for the use of PGS in clinical practice, confirming the value of identifying high-risk individuals for CAD development that may benefit from early intervention in terms of lifestyle changes and as a preventative therapeutic approach.

## Figures and Tables

**Table 1:** Study population description by main features correlated with coronary artery disease. In the EPICOR cohort, these parameters were registered at the enrolment; all the individuals in the pre-clinical CAD group had a MI during the study follow-up. *p*-values were obtained by the Mann–Whitney test for continuous variables and by the Fisher test for categorical variables; * indicates statistical significance (* < 0.05, ** < 0.01, *** < 0.001). Abbreviations: SD = standard deviation; BMI = body mass index; HDL = high-density lipoprotein; LDL = low-density lipoprotein; PAS = systolic arterial pressure; PAD = diastolic arterial pressure.

Characteristics		EPICOR Study (576 Individuals)			ATVB Study (3359 Individuals)		
	Unit	Pre-clinical CAD individuals (286)	Disease-free individuals (290)	*p*-value	CAD Patients (1691)	Disease-free individuals (1668)	*p*-value
Age	years mean (SD)	53 (±7.3)	53 (±7.5)	0.94	40 (±4.9)	40 (±4.9)	0.752
Sex							
Male		184	186	1.0	1498	1474	0.871
Female		102	104		193	194	
BMI	kg/m^2^ mean (SD)	27 (±3.7)	26 (±3.9)	0.0002 ***	27 (±4.2)	25 (±3.3)	<0.0001 ***
Total Cholesterol	mmol/L mean (SD)	6.2 (±1.2)	6.0 (±1.2)	0.20	5.7 (±1.4)	5.2 (±1.0)	<0.0001 ***
Hypercholesterolemia		236	192	0.015 *	933	690	<0.0001 ***
HDL	mmol/L mean (SD)	1.4 (±0.4)	1.6 (±0.4)	<0.0001 ***	1.1 (±0.3)	1.3 (±0.3)	<0.0001 ***
LDL	mmol/L mean (SD)	4.0 (±1.0)	3.7 (±1.0)	0.012 *	3.7 (±1.4)	3.2 (±0.9)	<0.0001 ***
Triglycerides	mmol/L mean (SD)	1.8 (±1.2)	1.6 (±0.9)	0.016 *	2.0 (±1.5)	1.3 (±0.8)	<0.0001 ***
Glycaemia	mmol/L mean (SD)	5.8 (±1.8)	5.5 (±1.0)	0.166	6.2 (±2.2)	5.0 (±0.8)	<0.0001 ***
Diabetes		7	2	0.105	131	14	<0.0001 ***
Hypertension		122	96	0.03 *	459	148	<0.0001 ***
PAS	mmHg mean (SD)	140 (±19)	136 (±19)	0.008 **	132 (±21)	124 (±14)	<0.0001 ***
PAD	mmHg mean (SD)	86 (±9)	85 (±11)	0.427	83 (±14)	82 (±41)	<0.0001 ***
Smoke							
Yes		121	68	<0.0001 ***	709	294	<0.0001 ***
No		86	123	(reference)	220	527	(reference)
Former		79	99	0.54	758	845	<0.0001 ***

**Table 2:** PRS distributions differences across Italian geographical macro-areas. The Kolmogorov–Smirnov test result summary to check if there are significant differences in the score distribution between and within the Italian geographical macro-areas.

**Kolmogorov–Smirnov Test** **Macro-Area Comparison**	**PGS000010** **(*p*-value)**	**PGS000329** **(*p*-value)**	**PGS001355** **(*p*-value)**	**PGS003727** **(*p*-value)**	**PGS004595** **(*p*-value)**
North vs. Center	0.27	0.01	<0.0001	<0.0001	0.05
North vs. Sardinia	0.03	0.06	0.14	0.73	0.69
North vs. South	0.98	0.0004	<0.0001	<0.0001	0.49
Center vs. Sardinia	0.15	0.44	0.51	0.18	0.43
Center vs. South	0.29	0.85	0.02	0.13	0.44
Sardinia vs. South	0.03	0.26	0.90	0.50	0.64
**Kolmogorov–Smirnov Test** **Sex comparison (Female vs. Male)**	**PGS000010** **(*p*-value)**	**PGS000329** **(** ***p*-value)**	**PGS001355** **(*p*-value)**	**PGS003727** **(*p*-value)**	**PGS004595** **(*p*-value)**
North (230 vs. 1800)	0.98	0.18	0.35	0.13	0.43
Center (46 vs. 374)	0.67	0.73	0.43	0.21	0.37
Sardinia (14 vs. 54)	0.42	0.85	0.57	0.97	0.51
South (84 vs. 660)	0.91	0.54	0.61	0.16	0.35
**Kolmogorov–Smirnov Test** **Cases vs. Controls comparison**	**PGS000010** **(*p*-value)**	**PGS000329** **(*p*-value)**	**PGS001355** **(*p*-value)**	**PGS003727** **(*p*-value)**	**PGS004595** **(*p*-value)**
North (903 vs. 1127)	<0.0001	0	<0.0001	<0.0001	<0.0001
Center (269 vs. 151)	0.13	<0.0001	<0.0001	<0.0001	0.002
Sardinia (42 vs. 26)	0.12	0.02	0.007	0.0001	0.53
South (419 vs. 325)	0.0002	<0.0001	<0.0001	<0.0001	<0.0001

## Data Availability

No sequencing data were generated for this study. The datasets and code supporting the current study have not been deposited in a public repository because data are not public but are available from the corresponding author upon request and approval by the Data Access Committee.

## Supplementary Materials

The following supporting information can be downloaded at: https://www.mdpi.com/article/10.3390/jpm14101025/s1. Figure S1: Polygenic risk score distributions. Figure S2: Principal component analyses. Figure S3: PRS distribution differences within geographical Italian macro-areas. Table S1: Selected PGS. Summary of the main characteristics for each PGS selected for the validation in the two large Italian cohorts. Table S2: Main results in the EPICOR study. Summary of the main characteristics for each score and main results of PRS calculation and statistical analyses in the EPICOR study. Table S3: Main results of the ATVB study. Summary of the main characteristics for each score and main results of PRS calculation and statistical analyses in the EPICOR study. Table S4: Geographical area description by main features correlated with coronary artery disease. Table S5: PRSs Comparison in the geographical areas. The AUC and their 95% confidence interval of the model based on the PRS were reported for the different areas of Italian peninsula. Abbreviations: AUC = area under the ROC curve, 95% CI = confidence interval of 95%. Supplementary methods regarding data imputation and QC, PRS calculation, geographical origin, outcomes, and definitions of variables. Description of the supplementary file: ResultsTable.xlsx.
