# Supplementary material for: Population Heterogeneity and Selection of Coronary Artery Disease Polygenic Scores
Source: J Pers Med. 2024 Sep 26;14(10):1025. doi: 10.3390/jpm14101025 (PMC11508882; doi:10.3390/jpm14101025)
Supplement: Supplementary file 1 [file jpm-14-01025-s001.zip › jpm-3209584-supplementary.pdf]

Population heterogeneity and selection of Coronary Artery Disease polygenic scores: an Italian example.

Carla Debernardi[1], Angelo Savoca[1], Alessandro De Gregorio[1], Elisabetta Casalone[1], Miriam Rosselli[1], Elton Jalis Herman[1], Cecilia Di Primio[1], Rosario Tumino[2], Sabina Sieri[3], Paolo Vineis[4], Salvatore Panico[5], Carlotta Sacerdote[6], Diego Ardissino[7-8], Rosanna Asselta[9-10], Giuseppe Matullo*[1-11].

SUPPLEMENTARY MATERIALS:

Supplementary Methods:

Data Imputation and QC

Data imputation was made through the Michigan Imputation server31, using the Genome Reference Consortium Human Build 37, r1.1 2016 as reference panel. Data pre-processing was made aligning the data to the reference build from 1000 Genomes Project and dbSNP (b151) by Plink 1.9 (http://pngu.mgh.harvard.edu/purcell/plink/) and BCFtools32.

We filtered data after imputation considering as criteria a minor allele frequency (MAF) < 0.01 and an R2 > 0.6, leaving 7,080,677 variants for EPICOR, and 7,263,254 variants for the ATVB dataset. Then for each PGS we calculated the percentage of SNPs with a good imputation score (R2 > 0.8), to check how reliable the polygenic score could be.

PRS Calculation

As form the definition of polygenic risk scores, we calculated each score as a sum of genome-wide genotypes, weighted by corresponding genotype effect size estimates, produced by the authors of the PGS.

We took into consideration the allele inversions in our genomic data and proceeded with a flipping (when necessary) to calculate the scores. For the calculation, we used PLINK 1.9 by the function score.

Geographical Origin

Study participants reported their province of birth on the enrollment questionnaire. There were only 8 missing data and 43 individuals were born outside Italy. For the remaining 3308, principal component (PC) analysis was carried out, in order to confirm their geographical origin. We removed 6 individuals, because their PCs had values that were very distant from the rest of the cohort. PCs Plot reflected the geographical subgroups (Supplementary Figure 2).

Outcome definition

In this study, we considered as CAD incident cases individuals with a fatal and nonfatal events of myocardial infarction.

Myocardial infarction events were noted on the records, backed up by information on symptoms at onset, it was defined as resting chest pain lasting >30 minutes accompanied by ST-segment elevation evolving into pathological Q waves and was confirmed by the presence of cardiac enzymes and troponins levels.

Anthropometric measures

Trained nurses following procedures settled in the protocols collected anthropometric measurements for weight, height, and BMI.

Blood pressure

Systolic (PAS) and diastolic (PAD) blood pressure measurements were conducted by specifically trained operators with the use of a mercury sphygmomanometer following standardized procedures. Subjects with a systolic blood pressure ≥140 mm Hg and/or a diastolic blood pressure ≥90 mm Hg (17) or reporting a clinical diagnosis of hypertension were considered hypertensive.

Blood Lipids levels

Each participant donated a blood sample from which they were measured according to standards: total cholesterol, HDL, triglycerides and glycaemia levels.

LDL was calculated by FF (total cholesterol (TC) minus high-density lipoprotein (HDL)-cholesterol minus triglycerides (TGs)/5 in mg/dl).

Hypercholesterolemia was defined as a fasting total serum cholesterol level of >200 mg/dL (5.2 mmol/L).

Other Variables

Participants reporting in a questionnaire: history of diabetes, smoking habits, coffee and alcohol consumption.

Supplementary Files:

ResultsTable.xlsx

This exel file contains 4 sheets:

1. the first contains the results of the main tests for all the PGSs available in the PGS Catalog (266)
2. the second sheet contains only the data of the PGSs that appear to have good performance (104)
3. the third sheet contains the data of the PGSs that result have good performances selected for the EFO code EFO_0001645 (49)
4. the fourth sheet contains only the PGS trained and tested with samples with a European ancestry (5)

Supplementary Figures:

Figure S1: Polygenic Risk Scores distributions. The first line of graphs showed in EPICOR study and the second line in ATVB study. PGS000010, PGS000329, PGS001355, PGS003727, PGS004595 scores distribution divided between pre-diagnostic CAD (cases) and disease free individuals (controls).

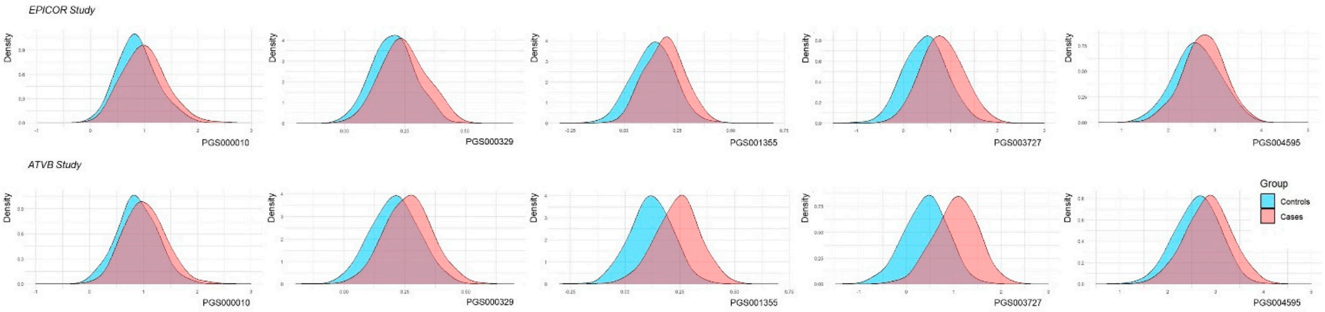

Figure S2: Principal Component Analyses. A) First two principal component plot colored to discriminate the geographical Italian macroareas (North, Centre, Sardinia and South). X axe was inverted to emphasize similarity to the geographical map of Italy. B) In black EPICOR individuals overlapped with ATVB individuals

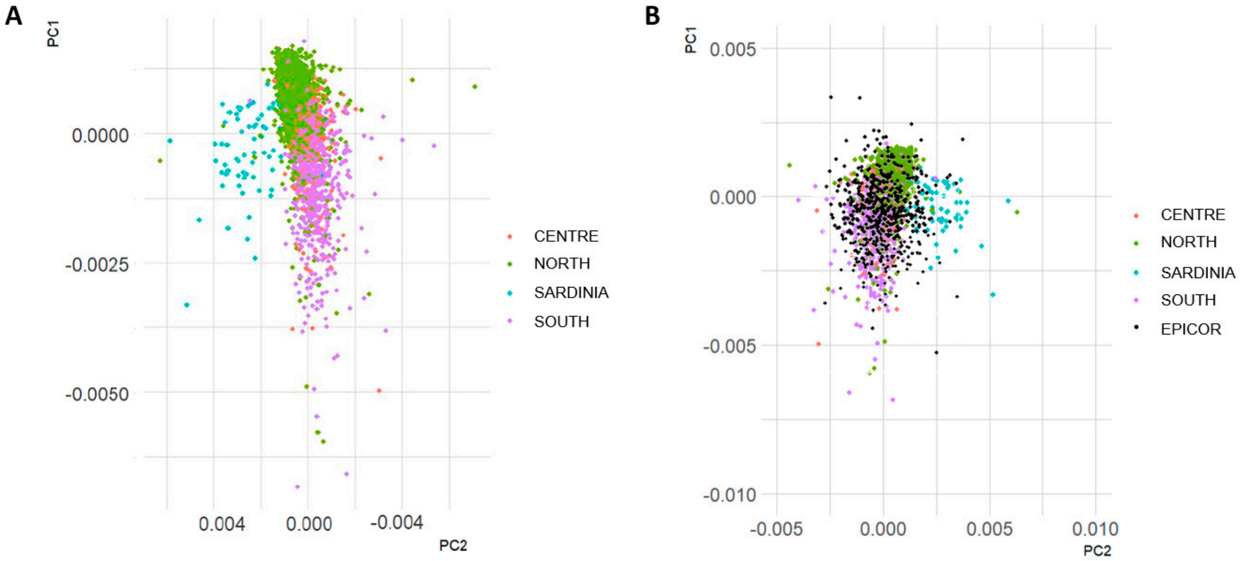

Figure S3: PRSs distribution differences within geographical Italian macro-areas. These Violin plots showed the scores distributions divided for PGS. On the left side of the violin, the distributions for the disease-free individuals and on the right the scores distributions for the patients with a myocardial infarction.

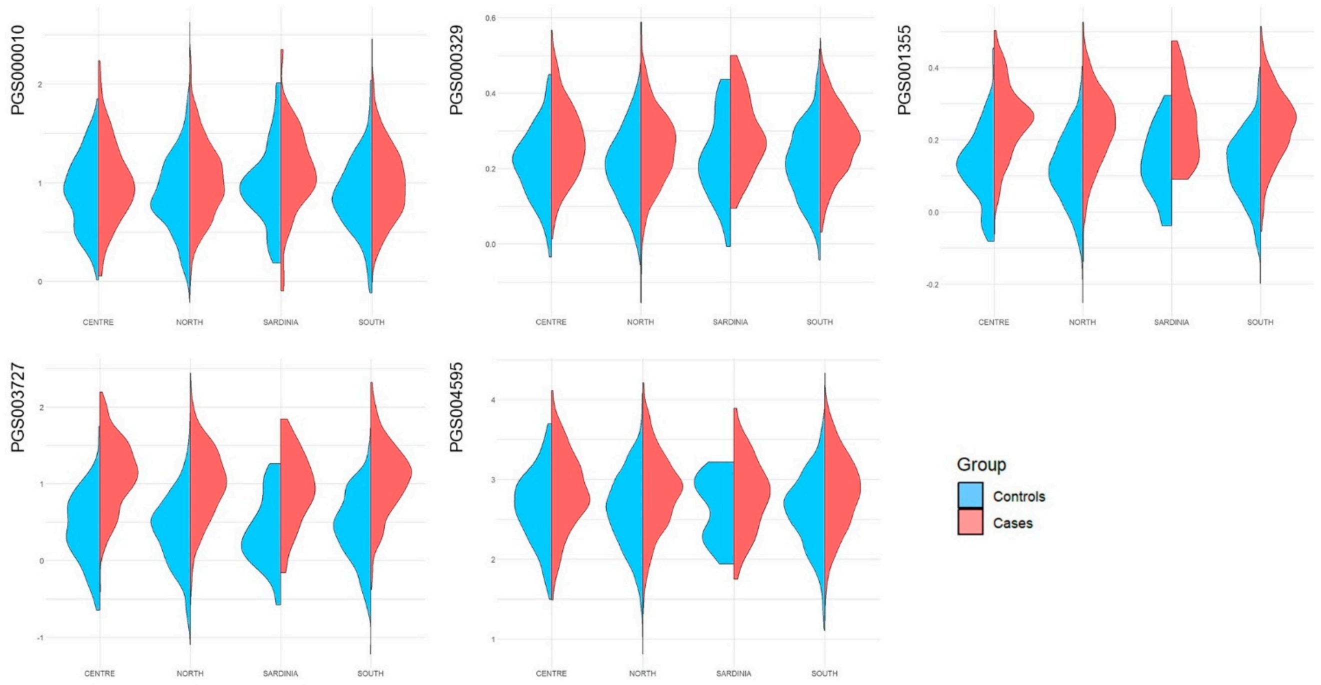

Supplementary Tables:

Table S1: Selected PGS. Summary of the main characteristics for each PGS selected for the validation in the two large Italian cohorts. Samples column indicates the number of samples used for the GWAS as source of variant associations. European column indicates the percentage of European individuals used in the samples column.

PGS	***Traits***	***EFO code***	***N° variants***	***Samples***	***European***	***Publication***
PGS000010	Coronary heart disease	EFO_0001645	27	86995	100%	Mega JL et.al, 2015 [27]
PGS000329	Coronary heart disease	EFO_0001645	6423165	408458	100%	Mars N et.al, 2020 [28]
PGS001355	Coronary artery disease	EFO_0001645	2994055	184305	100%	Ye Y et.al, 2021 [29]
PGS003727	Coronary artery disease	EFO_0001645	1125113	504636	100%	Patel AP et.al, 2023 [30]
PGS004595	Coronary heart disease	EFO_0001645	164	773268	100%	Oni-Orisan A et.al, 2022 [31]

Table S2: Main Results in EPICOR Study. Summary of the main characteristics for each score and main results of PRS calculation and statistical analyses in the EPICOR Study.
*General Linear Model (GLM) to determine if there is a statistically significant association between having had a CAD event and PRS
** Odds Ratio adjusted for age, sex and BMI for the individuals whose score fell beyond a certain threshold.
***DeLong's Test to demonstrate if adding PRS to the covariates model (AUC = 0.71; 95% CI = 0.60-0.81) could improve significantly the performance on the test set.

EPICOR Study	***PGS000010***	***PGS000329***	***PGS001355***	***PGS003727***	***PGS004595***
EFO code	EFO_0001645	EFO_0001645	EFO_0001645	EFO_0001645	EFO_0001645
N° variants	27	6423165	2994055	1125113	164
Coverage (%)	81.48%	97.41%	99.78%	99.96%	77.44%
KS Test (p-value)	0.000129	6.48E-06	1.90E-08	2.80E-14	0.001507
Wilcox Test (p-value)	2.72E-05	3.62E-08	1.84E-10	2.82E-19	0.00024
GLM (p-value)*	2.45E-05	7.37E-09	3.56E-11	7.47E-21	0.00029
*OR** score > 80th (95% CI)*	1.98 (1.30-3.05)	2.30 (1.51-3.57)	2.82 (1.83-4.43)	4.30 (2.72-6.98)	1.14 (0.75-1.72)
*OR** score > 90th (95% CI)*	1.71 (0.98-3.05)	2.91 (1.62-5.47)	2.59 (1.45-4.81)	6.54 (3.28-14.55)	1.31 (0.76-2.28)
*OR** score > 95th (95% CI)*	2.26 (1.03-5.33)	7.07 (2.68-24.34)	3.22 (1.41-8.30)	7.08 (2.69-24.37)	1.31 (0.61-2.84)
AUC ROC model PRS (95% CI)	0.54 (0.44-0.65)	0.66 (0.56-0.76)	0.64 (0.53-0.74)	0.68 (0.59-0.78)	0.60 (0.49-0.70)
AUC ROC model PRS and covariates (95% CI)	0.69 (0.59-0.80)	0.74 (0.64-0.83)	0.73 (0.63-0.83)	0.76 (0.66-0.85)	0.72 (0.62-0.82)
*DeLong's Test*** (p-value)*	0.57	0.25	0.47	0.23	0.49

Table S3: Main Results in ATVB Study. Summary of the main characteristics for each score and main results of PRS calculation and statistical analyses in the EPICOR Study.
*General Linear Model (GLM) to determine if there is a statistically significant association between having had a CAD event and PRS
** Odds Ratio adjusted for age, sex and BMI for the individuals whose score fell beyond a certain threshold.
***DeLong's Test to demonstrate if adding PRS to the covariates model (AUC = 0.71; 95% CI = 0.60-0.81) could improve significantly the performance on the test set.

ATVB Study	***PGS000010***	***PGS000329***	***PGS001355***	***PGS003727***	***PGS004595***
EFO code	EFO_0001645	EFO_0001645	EFO_0001645	EFO_0001645	EFO_0001645
N° variants	27	6,423,165	2,994,055	1,125,113	164
Coverage (%)	81.48%	97.41%	99.78%	99.96%	77.44%
KS-Test (p-value)	1.11E-16	0	0	0	0
Wilcox Test (p-value)	6.61E-23	1.80E-55	8.14E-208	1.19E-250	3.48E-42
GLM (p-value)*	1.45E-25	4.67E-59	5.53E-230	4.61E-288	2.61E-44
*OR** score > 80th (95% CI)*	2.02 (1.69-2.43)	2.43 (2.02-2.93)	12.09 (9.38-15.81)	20.04 (13-63-30.82)	2.52 (2.10-3.04)
*OR** score > 90th (95% CI)*	2.46 (1.92-3.16)	2.93 (2.27-3.81)	11.64 (8.01-17.57)	23.10 (14.34-40.07)	3.00 (2.32-3.92)
*OR** score > 95th (95% CI)*	2.67 (1.90-3.81)	3.23 (2.25-4.72)	16.37 (9.03-33.41)	22.93 (11.52-54.32)	3.89 (2.67-5.82)
AUC ROC model PRS (95% CI)	0.57 (0.53-0.62)	0.65 (0.61-0.69)	0.78 (0.74-0.82)	0.80 (0.77-0.84)	0.59 (0.55-0.64)
AUC ROC model PRS and covariates (95% CI)	0.78 (0.74-0.82)	0.80 (0.76-0.84)	0.84 (0.80-0.87)	0.85 (0.81-0.88)	0.77 (0.73-0.82)
*DeLong's Test*** (p-value)*	0.33	0.17	0.002	0.0003	0.19

Table S4: Geographical areas description by main features correlated with Coronary Artery Disease. Abbreviations: SD = standard deviation, BMI = body mass index, HDL = high-density lipoprotein, LDL = low-density lipoprotein, PAS = systolic arterial pressure, PAD = diastolic arterial pressure.

Characteristics	***unit***	***North (N=2030)***	***Centre (N=420)***	***Sardinia (N=68)***	***South (N=744)***
Cardiovascular Disease (%)		903 (44.5%)	269 (64.0%)	42 (61.8%)	419 (56.3%)
Age	*years mean (SD)*	39.5 (±4.9)	39.8 (±4.9)	40.2 (±4.8)	39.8 (±4.8)
Sex					
Male (%)		1800 (89%)	374 (88%)	54 (79%)	660 (89%)
Female (%)		230 (11%)	46 (12%)	14 (21%)	84 (11%)
BMI	*Kg/m2 mean (SD)*	25.5 (±3.8)	26.4 (±3.8)	24.7 (±2.8)	26.6 (±3.9)
Total Cholesterol	*mmol/l mean (SD)*	5.5 (±1.2)	5.5 (±1.3)	5.1 (±1.3)	5.5 (±1.3)
Hypercholesterolemia (%)		976 (48%)	210 (50%)	28 (41%)	382 (49%)
HDL	*mmol/l mean (SD)*	1.2 (±0.3)	1.1 (±0.0)	1.2 (±0.3)	1.1 (±0.3)
LDL	*mmol/l mean (SD)*	3.5 (±1.2)	3.5 (±1.1)	3.1 (±1.2)	3.5 (±1.2)
Triglycerides	*mmol/l mean (SD)*	1.6 (±1.2)	1.8 (±1.1)	1.7 (±1.3)	1.8 (±1.2)
Glycaemia	*mmol/l mean (SD)*	5.5 (±1.6)	5.8 (±1.7)	5.8 (±1.5)	5.8 (±2.1)
Diabetes (%)		71 (3%)	22 (5%)	3 (4%)	44(6%)
Hypertension (%)		356 (18%)	91 (22%)	13 (19%)	127 (17%)
PAS	*mmHg mean (SD)*	130 (±19)	127 (±18)	131 (±18)	128 (±20)
PAD	*mmHg mean (SD)*	84 (±36)	81 (±12)	82 (±9)	82 (±13)
Smoke					
Yes (%)		707 (35%)	170(40%)	28 (41%)	336 (44%)
No (%)		758 (37%)	91 (22%)	15 (22%)	176 (24%)
Former (%)		561 (28%)	158 (37%)	25 (37%)	232 (32%)

Table S5: PRSs Comparison in the geographical areas. The AUC and their 95% confidence interval of the model based on the PRS were reported for the different areas of Italian peninsula. Abbreviations: AUC = area under the ROC curve, 95% CI = confidence interval of 95%.

PGS	*AUC (95%CI)*	***North (N=2030)***	***Centre (N=420)***	***Sardinia (N=68)***	***South (N=744)***
PGS000010		0.64 (0.59-0.69)	0.59 (0.46-0.72)	0.85 (0.55-1)	0.55 (0.46-0.64)
PGS000329		0.66 (0.61-0.72)	0.66 (0.53-0.79)	0.62 (0.27-0.98)	0.66 (0.58-0.75)
PGS001355		0.81 (0.76-0.85)	0.79 (0.68-0.90)	0.87 (0.67-1)	0.78 (0.71-0.86)
PGS003727		0.82 (0.78-0.86)	0.82 (0.72-0.92)	0.97 (0.91-1)	0.83 (0.76-0.89)
PGS004595		0.63 (0.57-0.68)	0.68 (0.56-0.81)	0.80 (0.54-1)	0.66 (0.57-0.74)
